# Mental health literacy and help-seeking: the mediating role of self-stigma and emotional intelligence

**DOI:** 10.3389/fpsyg.2025.1589093

**Published:** 2025-08-14

**Authors:** Mahd Awan, Melissa A. Boyce, Brittany L. Lindsay

**Affiliations:** Department of Psychology, University of Calgary, Calgary, AB, Canada

**Keywords:** mental health literacy, help-seeking, stigma, emotional intelligence, mental health

## Abstract

When faced with mental health concerns, help-seeking can be a useful means to seek and receive help from formal support sources—such as mental health professionals, as well as informal support sources—such as friends and family. Both the intention and tendency to engage in formal help-seeking are predicted by mental health literacy, self-stigma, and emotional intelligence; however, the role that each of these factors play in relation to informal help-seeking is less clear. The current study examined the predictive value of mental health literacy with respect to both formal and informal help-seeking intentions. Additionally, the current study explored the role of self-stigma and emotional intelligence as possible mediators of these relationships. Undergraduate students (*n* = 301) were recruited from a Western Canadian university and completed a series of online questionnaires measuring their formal and informal help-seeking intentions, mental health literacy, self-stigma faced when seeking help, and meta-mood, as an operationalization of emotional intelligence. Results indicated that mental health literacy was a significant positive predictor of formal help-seeking intentions, and that both self-stigma and meta-mood partially mediated this relationship. Furthermore, results showed that mental health literacy did not serve as a significant predictor of informal help-seeking, although mental health literacy did have a significant indirect effect on informal help-seeking, through the mediation of meta-mood. The importance of self-stigma and meta-mood in relation to mental health literacy are highlighted in terms of formal help-seeking outcomes, and the implications of these findings for informal help-seeking are discussed.

## 1 Introduction

In Canada, it is estimated that the lifetime prevalence of experiencing at least one mental illness is 50% by the age of 40 (Smetanin et al., 2011). Despite the large proportion of individuals facing mental health concerns, the use of formal mental health resources by Canadians is limited. For example, Stephenson (2023) found that only 16.8% of surveyed Canadians had reached out to a medical professional in the past year regarding their mental health. Even among Canadians who met the diagnostic criteria for a mood or anxiety disorder, only about half had reached out to a medical professional in the past year for mental health support, despite findings that the majority of individuals who have accessed professional treatment had their needs met through counselling (64.3%), information (82.3%), or medication (92%) (Stephenson, 2023; Sunderland and Findlay, 2013). Evidently, there exists a gap between those who need mental health support and those who access and receive professional mental health support (i.e., a treatment gap; Kohn et al., 2018; Keyes, 2007; Kohn et al., 2004).

This treatment gap is common among mental healthcare systems in North America which are underfunded and over-capacity (Knaak et al., 2017; Moroz et al., 2020; American Psychological Association, 2022). Nonetheless, proactively addressing known risk factors and promoting positive mental health in our communities can help reduce the burden on the healthcare system by preventing the onset of higher severity disorders (Gilmour, 2014; Kisling and Das, 2023). Although professional treatments are considered a quintessential method of supporting one’s mental health, informal social support may present a more accessible way to reduce the risk of developing harmful mental health outcomes; social support often serves as a protective factor against the onset and severity of many mental illnesses, as well as improving positive mental health outcomes and well-being (Levula et al., 2016; Liu et al., 2021; Southwick et al., 2005). For example, Dong et al. (2024) investigated the benefits of informal support for older adults and found that increased levels of contact and support from one’s children was a strong predictor of better mental health outcomes. A review from Lynch et al. (2023) found that informal support can either facilitate or hinder mental health outcomes and formal help-seeking, depending on the effectiveness of the support network and the quality of the interactions within it. These findings highlight that not all social support is helpful – its impact depends on the nature of the interactions rather than the amount of support alone. Therefore, understanding support from the perspective of the help-seeker is critical for accurately capturing the help-seeking experience and designing effective interventions. A comprehensive psychosocial framework should consider both individual experiences and environmental factors affecting support seeking (Biddle et al., 2007; Holden et al., 2012; Carpentier and Bernard, 2011; Nash et al., 2017). In the current study, considering that individuals may be relying more on social supports as a response to barriers in formal support utilization, it is important for research on help-seeking behaviors to parse *formal help-seeking*, such as therapy or treatment delivered by mental health professionals, and *informal help-seeking*, often provided by an individual’s personal connections.

Generally, help-seeking is a means to receive support and address support needs in response to emotional, behavioral, or cognitive concerns that threaten the individual’s wellbeing (Rickwood and Thomas, 2012; Leamy et al., 2011; Tew et al., 2012). In response to a mental health concern, individuals can seek out *formal* supports, which are often a paid professional service (Elkin et al., 1995). These resources may be more suited for the diagnosis and treatment of mental health symptoms and distress, including specific evidence-based interventions for mental illnesses and promotion of positive mental health (Elkin et al., 1995; Lauzier-Jobin and Houle, 2022). Mental health professionals vary in their effectiveness, which can be influenced by the alignment between the client’s specific area of concern and needs, types of treatment provided, competency of the professional, and available resources (Andrews, 1999; Elkin et al., 1995; Stephenson, 2023; Brewer et al., 2024). Despite this variability, most individuals within Canada who accessed formal supports felt that their mental health concerns were fully addressed (Stephenson, 2023), underscoring the value of formal supports in helping to address mental health concerns.

In contrast, informal help-seeking often refers to help sought through personal connections and can include friends, family, partners, and peers (Rickwood and Thomas, 2012). According to Lauzier-Jobin and Houle (2022), these personal supports tend to be less prepared for—and less knowledgeable about – how best to support individuals with specific mental health challenges. Despite this lack of formal training, informal support can provide emotional closeness, a sense of belonging, and a personal bond that may not be present in formal supports. Furthermore, informal helpers tend to have a pre-existing relationship with those that they help. In this dynamic there is higher reciprocity in help given and received, in addition to being more accessible than formal supports. Informal supports are, however, limited in the type of support they can reasonably provide, as they often do not have relevant training to address mental health concerns. Despite this, informal support has been associated with a general reduction in the odds of suffering from psychological distress, especially after experiencing distressing life events (Maulik et al., 2010; Scott et al., 2020). It is also associated with reduced symptoms of depression and post-traumatic stress disorder (Bryant et al., 2017). Following an intervention for subclinical depressive symptoms, Yiu et al. (2025) found that emotional support from formal sources served as a greater predictor of reductions in depressive symptoms, whereas emotional support from informal sources served as a predictor of decreases in anxiety symptoms. While the methodology limits conclusions about how formal and informal support differentially benefited participants based on symptomology, the study highlights the distinct effects of each support type. The literature has identified the utility of peer support, specifically, which has been shown to be associated with increases in effective coping, self-reported recovery, and self-empowerment (Richard et al., 2022; White et al., 2020). A deeper understanding of peer support and other informal support mechanisms may help translate theoretical and empirical insights into a comprehensive operational framework to inform intervention implementation and use (Rickwood and Thomas, 2012).

While formal help-seeking has been established as one of the primary methods of addressing mental health concerns (Elkin et al., 1995), accessibility of formal supports complicates utilization. Significant barriers, such as affordability, cultural beliefs towards support, knowledge of supports, and ease of access and availability, can all inhibit formal help-seeking behaviors and intentions (Carbonell et al., 2020; Eisenberg et al., 2011; Guo et al., 2015; Moroz et al., 2020; Planey et al., 2019; Zimmerman, 2005). These same barriers, however, do not necessarily inhibit informal help-seeking, such as talking to a trusted friend (Saltzman et al., 2020). Additionally, the structures, goals, and expectations associated with formal supports are markedly different than those associated with informal supports (Lauzier-Jobin and Houle, 2022). In formal contexts, support is usually goal-directed, in which the helper is deliberate and plans their actions, interacting with the help-seeker in a way that facilitates progress towards a certain pre-determined outcome (e.g., reducing the severity of anxiety symptoms; Lauzier-Jobin and Houle, 2022). Thus, the structure of formal support centers around how to reach a certain agreed-upon outcome. In contrast, a mutual goal is not necessarily present in informal help-seeking. Hence, while formal help-seeking is the prototypical conceptualization of help-seeking for mental health concerns, this position may disregard the importance of informal help-seeking, as well as the differences, appropriateness, and value across different situations in which an individual would benefit from informal support (Rickwood and Thomas, 2012). Because of the limited understanding of informal support in the literature (Heerde and Hemphill, 2018), the need for further exploration of informal support as its own construct is evident.

Despite these key differences between informal and formal supports, the relationship between these two types of support can be complicated. Fonseca and Canavarro (2017) found that informal support predicted increases in formal help-seeking, with male partner support predicting higher formal help-seeking intentions in pregnant female partners. However, while they are not necessarily substitutive, there is some indication that informal help-seeking can serve as a barrier to formal help-seeking. This occurs when informal support is perceived to be a sufficient replacement for formal support, or when social support reduces stress and consequently, the perceived need for formal support (Maulik et al., 2011). However, Maulik et al. (2011) found that increased contact with informal supports can often predict the use of more formal supports, which they refer to as a *referral function.* Despite the complex relationship, formal and informal supports are often used together (see Brown et al., 2014), and very few individuals engage in *only* formal help-seeking (D'Avanzo et al., 2012). Differences in support utilization are based on the severity and type of mental health concern, alongside other environmental and personal factors (Maulik et al., 2011; Brown et al., 2014). Due to the differences between the dynamics of formal and informal help-seeking, it may be useful to consider them as separate help-seeking types altogether (Oliver et al., 1999). Although informal and formal help-seeking can work together, it is necessary to also understand in what ways each type of help-seeking is distinct, such as the nature of the relationship between the support recipient and the support provider, to develop a richer functional understanding of each (Lauzier-Jobin and Houle, 2022; Lien et al., 2024; Lynch et al., 2023).

The likelihood of engaging in help-seeking behaviors can be influenced by a variety of factors, including intrapersonal differences in the person engaging (or not) in help-seeking behaviors. One important factor is Mental Health Literacy (MHL), or the knowledge and beliefs an individual possesses about mental health and illness (Iswanto and Ayubi, 2023; Jorm et al., 1997; Jorm, 2012; Lien et al., 2024). This includes knowledge of positive mental health, mental disorders, stigma towards mental illnesses, mental health supports, risk factors, coping strategies, and attitudes towards mental health and help-seeking (Jorm et al., 1997; Kutcher et al., 2016; Wei et al., 2015). MHL has been found to explain a significant amount of the variance in adolescents’ mental wellbeing (Bjørnsen et al., 2019) and is an important predictor in formal help-seeking (Murray and Knudson, 2023; Iswanto and Ayubi, 2023; Jung et al., 2017; O'Connor and Casey, 2015). Furthermore, a lack of MHL acts as a significant barrier to help-seeking (Bonabi et al., 2016; Gulliver et al., 2010; McCann et al., 2016).

While the link between MHL and formal or general help-seeking is well established (Iswanto and Ayubi, 2023; Lien et al., 2024), the relationship between MHL and informal help-seeking has yet to be clearly established in the literature (Heerde and Hemphill, 2018). For example, Smith and Shochet (2011) found that MHL significantly explained the variance across general help-seeking intentions, which was operationalized to include both formal and informal supports. However, a similarly structured study by Samar and Perveen (2021), which used a combined general help-seeking outcome, did not find a relationship between MHL and help-seeking. Increases in MHL, however, have been shown to be associated with discussion of mental health concerns in general (Lindow et al., 2020). More broadly, Lien et al. (2024) found MHL to be a key factor influencing help-seeking attitudes while recognizing the importance of multiple other factors. These findings highlight the need for a standardized approach, as the field lacks a theoretical framework that fully captures the potential differences between formal and informal support. It is therefore critical to understand the mechanisms underlying the relationship between MHL and both formal *and* informal help-seeking to provide a comprehensive understanding of this key area for intervention for both types of support (Fairchild et al., 2009).

Two other intrapersonal factors may be important to consider in the relationship between MHL and help-seeking behaviors: stigma and emotional intelligence. Stigma is an overarching term for the process of negatively labelling human differences within our society (e.g., mental illnesses), causing a variety of negative outcomes for those with these labels including negative stereotypes, separation, and status loss (Link and Phelan, 2001). Although public (social) stigma, which exists at the interpersonal level, is a common area of research, stigma can also exist at the structural and intrapersonal levels (Knaak et al., 2017; Livingston, 2020). Stigma at the intrapersonal level is called internalized stigma or self-stigma, and can specifically include negative beliefs (stereotypes), attitudes (prejudice), and behaviours (discrimination) toward help-seeking (Corrigan and Rao, 2012). Self-stigma is the internalization of structural and public stigma whereby an individual devalues themselves based on perceived markers of social distinction (Mak et al., 2007) and can be associated with a decrease in self-esteem and the denigration of one’s self concept (Corrigan et al., 2006).

Past literature has recognized stigma toward help-seeking as a key barrier to formal help-seeking intentions and behaviors, highlighting that both external and self-stigma can be difficult for individuals to overcome (Corrigan et al., 2006; Vogel et al., 2006). Cawley-Fiset (2016) found that as MHL increased, self-stigma of help-seeking decreased, and positive attitudes toward formal help-seeking increased—indicating that self-stigma may be a pathway through which MHL influences help-seeking (Lannin et al., 2016). With greater knowledge of one’s mental health challenges, one is better able to understand the benefits of seeking support to address those challenges. Although the literature is limited, mental health stigma has also been shown to be related to informal help-seeking and MHL (Lindow et al., 2020). However, Jung et al. (2017) found that while MHL predicted attitudes towards help-seeking, neither self-stigma, nor externalized mental health stigma served as mediators. They did find that MHL was a predictor of externally focused stigma; however, externally focused stigma was not a predictor of attitudes towards help-seeking. Conversely, they found MHL was not a predictor of self-stigma, although self-stigma was a predictor of attitudes towards help-seeking. Due to the lack of clarity regarding the role of self-stigma of help-seeking, there is a need for further research in this area (Tucker et al., 2013).

The second explanatory factor, emotional intelligence (EI), broadly refers to an individual’s ability to perceive, understand, regulate, and reflect on emotions (Mayer and Salovey, 1995; Mayer, 1997; Mayer et al., 2000). While some view EI as a cognitive ability, others conceptualize it as a as a trait encompassing well-being, self-regulation, emotionality and sociability (Hughes and Evans, 2018; Petrides et al., 2016; Petrides, 2009). Furthermore, some conceptualize EI as a combination of both perspectives (Bar-On, 1997; Goleman, 2005; Bru-Luna et al., 2021). While research directly examining EI and help-seeking—formal or informal—is limited, EI has been linked to instrumental and emotional social support-seeking, as well as positive mental health outcomes (Gohm and Clore, 2002; Sarrionandia and Mikolajczak, 2019). Furthermore, specific facets of EI, such as self-compassion and emotional openness have been linked to favorable attitudes towards help-seeking (Dschaak et al., 2021; Kim et al., 2022; Komiya et al., 2000). Perception and understanding, regulation, and labelling are all identified facets of EI shown to be related to general help-seeking attitudes (Gecaite et al., 2016; Cuvalo, 2021). Similar concepts such as emotional competency (the capacity of an individual to perceive and regulate personal and others’ emotions) have also been shown to be associated with informal help-seeking from family and friends (Ciarrochi et al., 2003). The proposed mechanism behind these links is the capacity to which an individual can manage or perceive the management of their emotions and mood, which often serve as indicators of one’s mental state (Gross et al., 2019).

Salovey et al. (1995) describes meta-mood as a facet of EI that refers to individuals’ awareness of, attention to, and ability to repair their emotional state. Conceptually, meta-mood aligns with mental health literacy (MHL) when defined as the application of mental health knowledge to oneself—particularly in recognizing, understanding, and managing emotions. Although limited research has examined the specific link between meta-mood and help-seeking, the focus of meta-mood on emotional awareness, consideration, and regulation strongly maps onto key facets of MHL. Namely the recognition and consideration that one may be experiencing something concerning. Consequently, regulation may include help-seeking as a form of recovery from an emotional or mental health concern. This similarity may serve to partially explain the relationship between MHL and both formal and informal help-seeking (O'Connor and Casey, 2015; Gecaite et al., 2016; Fteiha and Awwad, 2020; Cuvalo, 2021), which is why it was selected as the operationalization of EI in the current study.

The current study investigated the relationships between MHL and formal and informal help-seeking intentions, in addition to examining self-stigma and EI (operationalized as meta-mood) as possible mediators of these relationships. To help delineate informal and formal help-seeking in the literature, and provide evidence for two possible explanatory variables, the current study utilized a cross-sectional survey design to investigate these relationships with two primary research aims: (1) Assess the relationship between MHL and formal and informal help-seeking intentions; and (2) Determine whether stigma towards help-seeking or meta-mood mediate this relationship. Consistent with previous literature (Smith and Shochet, 2011), the first hypothesis was that there would be a positive relationship between MHL and both formal (Hla) and informal (H1b) help-seeking intentions. The second hypothesis was that stigma towards help-seeking (H2a) and meta-mood (H2b) would mediate the relationship between MHL and *formal* help-seeking. Given the paucity of research on the relationship between MHL and *informal* help-seeking, no specific hypotheses were made regarding whether self-stigma or meta-mood would mediate this relationship. This aspect of the study was exploratory in nature.

## 2 Methods

### 2.1 Participants

Utilizing a convenience sample, a total of 322 participants were recruited through the Department of Psychology’s Research Participation System (RPS) at a Western Canadian postsecondary institution. Participants were compensated for their time with bonus course credit in an eligible undergraduate psychology course. On the post-survey consent check, seven participants requested not to release their data, and another 14 participants were excluded due to incomplete responses. The final sample contained 301 participants, of which 278 identified as female, 17 as male, and six participants identified as non-binary. The ages of participants ranged from 17 to 51 (*M* = 19.88, *SD* = 3.69). Self-reported ethnic backgrounds were 40.6% White, 23.6% Southeast Asian, 10.3% East Asian, 6.0% Mixed, 5.3% Black, 4.3% Filipino, 3.7% Arab and West Asian, 2.0% Latin American, 1.3% Indigenous, 1.3% Other, 1.0% Caribbean, and 0.7% unsure. Although participants’ degree programs were not collected for the current study, the pool that participants were drawn from was comprised of 13.6% Psychology majors, 30.3% other Arts majors, 24.2% Science majors, 8.3% Community Rehabilitation and Disability Studies majors, 7.8% Kinesiology majors, with the rest of the pool coming from Engineering, Social Work, Nursing, Education, and Open Studies. Participants were also asked about their history of mental health issues (diagnosed or undiagnosed). Around half of the sample (56.1%; *n* = 169) reported a history of mental health issues, 37.9% reported no history, and 6% preferred not to disclose. Participants who reported a history of mental health issues were then asked about their prior help-seeking behaviors. Over half of this subset of participants (57.4%) reported using both formal and informal support, 26% reported using only informal supports, 8.3% reported using only formal supports, and 8.3% preferred not to disclose.

### 2.2 Measures

#### 2.2.1 The mental help seeking intention scale

The Mental Help Seeking Intention Scale (MHSIS) (Hammer and Spiker, 2018) measures intentions to seek professional help for mental health concerns. Assessing help-seeking intentions enables researchers to predict future help-seeking behaviors, rather than using a retrospective measure of actual help-seeking, which can be prone to recall error, and allows for cross sectional study designs that predict help-seeking (Whyte, 2021). Research has found that formal help-seeking intentions are often closely linked to formal help-seeking behaviors (Kim and Hunter, 1993; Hammer and Spiker, 2018).

For the purposes of this study, a set of items were developed to assess help-seeking intentions from informal sources (e.g., friends, family, etc.) by adapting the MHSIS. This was done by replacing the start of the prompt, “I would intend to seek support from a mental health professional” with “I would intend to seek support from someone other than a mental health professional.” The adapted scale contains six items, three of which measure formal help-seeking intentions and three of which measure informal help-seeking intentions. Higher scores on the scale reflect higher help-seeking intentions. In addition to this adaptation, the response scale was adapted from a 7-point Likert scale measuring agreement with statements (*Strongly Disagree—Neutral—Strongly Agree*), to a forced-choice 6-point Likert scale (*Strongly Disagree—Strongly Agree*). As a binary construct, the intention to seek help maps on to agreement with the statements; however, both a neutral response and disagreement with the statements indicate a lack of that intention (Sheeran, 2002). As such, a neutral response in intentionality is not a valuable response for a binary understanding of intentionality. Retaining a Likert scale, however, ensures that both intensity and directionality of intention can be assessed.

The MHSIS was developed based on principles from the theory of planned behavior, which posits that multiple factors combine to predict behavioral intentions, including attitudes toward the behavior, expectations of others, and perceived control in performing the behavior (Ajzen, 1991). The original scale has been found to have a high level of predictive validity (Hammer and Spiker, 2018). Both the original and adapted scales displayed high reliability for formal help-seeking intentions (*α* = 0.96) and informal help-seeking intentions (*α =* 0.91) in the current study.

#### 2.2.2 Mental health literacy scale

The Mental Health Literacy Scale (MHLS) (O'Connor and Casey, 2015) measures multiple components of MHL, including: (1) the ability to identify features of specific mental illnesses; (2) knowledge of how and where to learn about mental health; (3) understanding of risk factors that contribute to mental health issues; (4) knowledge of self-treatment practices; (5) where and how to access professional help, as well as knowing what services they can provide; (6) attitudes that promote recognition of mental health issues; and (7) attitudes that influence help-seeking, including stigma against mental illness, help-seeking, and individuals who have mental illnesses (Jorm et al., 1997; Wei et al., 2015).

The MHLS asks participants to respond to several items based on 4-point Likert scales for likeliness (*Very Unlikely*—*Very Likely*) and helpfulness (*Very Unhelpful*—*Very Helpful*), as well as 5-point Likert scales for agreement (*Strongly Disagree—Strongly Agree*) and willingness (*Definitely Unwilling—Definitely Willing*). It produces a unidimensional score, where higher scores reflect higher mental health literacy. The scale has 35 items, 12 of which are reverse coded. Example items include “To what extent do you think it would be helpful for someone to improve their quality of sleep if they were having difficulties managing their emotions (e.g., becoming very anxious or depressed)” and the reverse-coded, “A mental illness is not a real medical illness.” The MHLS demonstrates strong psychometric properties, including high construct validity and high internal consistency (O'Connor and Casey, 2015). The scale displayed good reliability (*α* = 0.88) in the current study.

#### 2.2.3 Self-stigma of seeking help

Designed by Vogel et al. (2006), the Self-Stigma of Seeking Help Scale (SSOSH) measures the stigma that individuals apply to themselves when considering seeking psychological help. The SSOSH has 10 items, five of which are reverse coded. An example item is “My self-confidence would NOT be threatened if I sought professional help.” Responses are measured on a 7-point Likert scale where 1 corresponds to *Strongly Disagree* and 7 corresponds to *Strongly Agree*. Higher scores on the scale reflect higher levels of self-stigma. The SSOSH has been found to have very strong psychometric properties, including high construct, criterion, and predictive validity (Vogel et al., 2006), in addition to high reliability in the current study (*α =* 0.90).

#### 2.2.4 Trait-Meta mood scale

The Trait Meta-Mood Scale was originally developed by Salovey et al. (1995) to measure clarity of, attention to, and the tendency to repair one’s emotions and moods. Clarity is concerned with an understanding of how one is feeling, whereas attention refers to whether an individual cares to pay attention to their emotions and mood. Repair describes an individual’s capacity and willingness to engage in maintenance of positive moods and to repair negative moods. The original 48-item scale was shortened to 24 items by Fernandez-Berrocal et al. (2004), with each sub facet retaining high reliability. An example item from the scale is “I think my emotions and state of mind deserve to be paid attention to.” Responses were adapted from a 5-point to a 7-point Likert scale measuring agreement with each item (Strongly Disagree—Strongly Agree) to improve scale sensitivity (see Finstad, 2010). Higher mean scores on the scale reflect higher emotional awareness, understanding, and care for one’s own mood. In this study, the scale displayed high reliability (*α =* 0.89).

### 2.3 Procedure

Participants accessed the study on the online Qualtrics survey platform, from a location of their choice, using a computer or mobile device, and provided informed consent prior to completing the study measures. Once consent was provided, participants were given 1 week to complete the survey. Scale order within the survey was randomized, as were the items in each scale. After completing the questionnaires, participants were asked demographic information including their age, gender, ethnicity, whether they had a history of mental illness, and, if so, whether they had previously utilized any mental health supports. After completing these measures, participants were debriefed and thanked for their participation. They were then asked to consent to submit their data for inclusion in the current study, as well as in future studies. Human research ethics approval was obtained for this study prior to data collection.

## 3 Results

### 3.1 Preliminary analyses

Prior to conducting mediation analyses to test the hypotheses, each of the assumptions of mediation were assessed, namely, (1) linearity between the variables of interest; (2) lack of multicollinearity between the variables of interest; and (3) normality of all variables of interest (Fein et al., 2022). A visual inspection of scatterplots for all combinations of the variables of interest confirmed no obvious non-linear patterns between any of the variables of interest. Correlation coefficients were examined between all variables of interest and confirmed that there was no multicollinearity between any of the variables (all *r*’s < 0.50). Lastly, a visual inspection of normality probability plots for all variables confirmed that each variable met the assumption of normality supporting use of mediation analyses. The means, standard deviations, and the minimum and maximum scores of each of the study measures can be seen in Table 1. Mediation statistics including: standardized betas (*β*), unstandardized betas (*B*), t-scores (*t*), standard error scores (*SE_β_*, *SE_B_*), *p*-values (*p*), and 95% confidence intervals (*95% CI_β_*, *95% CI_B_*) are reported. All statistical analyses were completed using SPSS Statistics Software (Version 29), and mediation analyses were completed using Hayes PROCESS Macro for SPSS (Version 4.2; Hayes, 2018).

**Table 1:** Descriptive statistics for study variables.

Statistical notation	*N*	*M*	*SD*	*Min*	*Max*
Mental health literacy scale	301	3.73	0.38	2.66	4.46
Formal help-seeking	301	4.17	1.13	1.00	6.00
Informal help-seeking	301	3.79	1.13	1.00	6.00
Self-stigma of help-seeking	301	2.96	1.10	1.00	5.90
Meta-mood	301	4.91	0.74	2.29	6.71

### 3.2 MHL, formal help-seeking, and self-stigma

**Figure 1:**
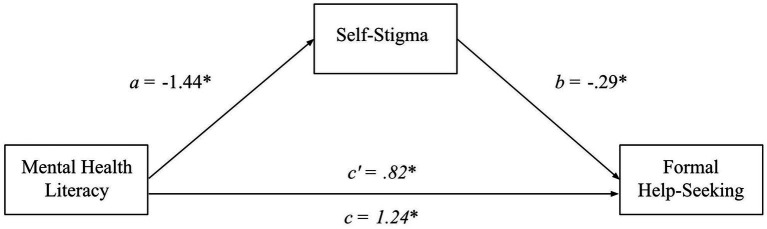
A mediation model of MHL, self-stigma, and formal help-seeking. This mediation model depicts formal help-seeking intentions as a function of MHL and self-stigma when self-stigma is controlled for (*c’*) and uncontrolled for (*c*). Furthermore, it shows the direct relationships of self-stigma to MHL and formal help-seeking. Lettered are the unstandardized beta coefficients for each relationship. *p < 0.001.

To determine whether there was a relationship between MHL and formal help-seeking, as well as if self-stigma mediated this relationship, a single-step mediation analysis was conducted (see Figure 1). The results revealed that MHL was a significant positive predictor of formal help-seeking (H1a supported), *β = 0.41, B = 1.24, t(299) = 7.80, SE_B_ = 0.16, p < 0.001*. Mental health literacy was also a significant negative predictor of the self-stigma of help-seeking, *β = −0.49, B = −1.44, t(299) = −9.82, SE_B_ = 0.15, p < 0.001*, and self-stigma of help-seeking was a significant negative predictor of formal help-seeking, *β = −0.28, B = −0.29, t(299) = −4.79, SE_B_ = 0.06, p < 0.001*. The indirect effect, bolstered by the bootstrap estimation (5,000 resamples), revealed self-stigma as a significant mediator of the relationship between MHL and formal help-seeking (H2a supported), (*β = 0.14, SE_β_ = 0.03, 95% CI_β_ = 0.07, 0.20; B = 0.41, SE_B_ = 0.10, 95% CI_B_ = 0.22, 0.62*). With the indirect effect controlled for, MHL remained a significant predictor of formal help-seeking (*β = 0.28, B = 0.82, t(299) = 4.67, SE_B_ = 0.18, 95% CI_B_ = 0.47, 1.17*), consistent with partial mediation.

### 3.3 MHL, formal help-seeking, and emotional intelligence (meta-mood)

**Figure 2:**
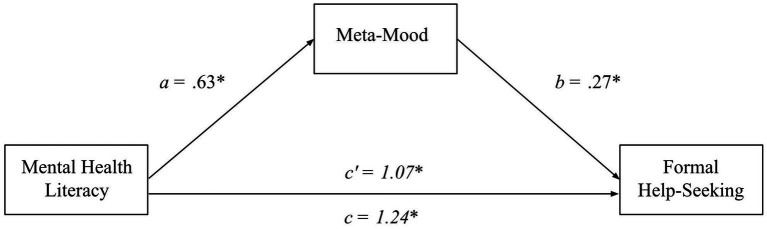
A mediation model of MHL, meta-mood, and formal help-seeking. This mediation model depicts formal help-seeking intentions as a function of MHL and meta-mood when meta-mood is controlled for (*c’*) and uncontrolled for (*c*). Furthermore, it shows the direct relationships of meta-mood to MHL and formal help-seeking. Lettered are the unstandardized beta coefficients for each relationship.

In addition to MHL being positively related to formal help-seeking as found in 3.2, the results showed that MHL was a significant positive predictor of meta-mood, *β = 0.31, B = 0.63, t(299) = 5.79, SE_B_ = 0.11, p < 0.001*, which was then a significant positive predictor of formal help-seeking, *β = 0.17, B = 0.26, t(299) = 3.16, SE_B_ = 0.08, p < 0.001.* The indirect effect, bolstered by the bootstrap estimation (5,000 resamples), revealed meta-mood as a mediator of the relationship between MHL and formal help-seeking (H2b supported; *β = 0.06, SE_β_ = 0.02, 95% CI_β_ = 0.02, 0.09; B = 0.17, SE_B_ = 0.06, 95% CI_B_ = 0.07, 0.29*). With the indirect effect controlled for, MHL remained a significant predictor of formal help-seeking, *β = 0.36, B = 1.07, t(299) = 6.50, SE_B_ = 0.17, 95% CI_B_ = 0.75, 1.40*, consistent with partial mediation; see Figure 2.

### 3.4 MHL, informal help-seeking, and self-stigma

**Figure 3:**
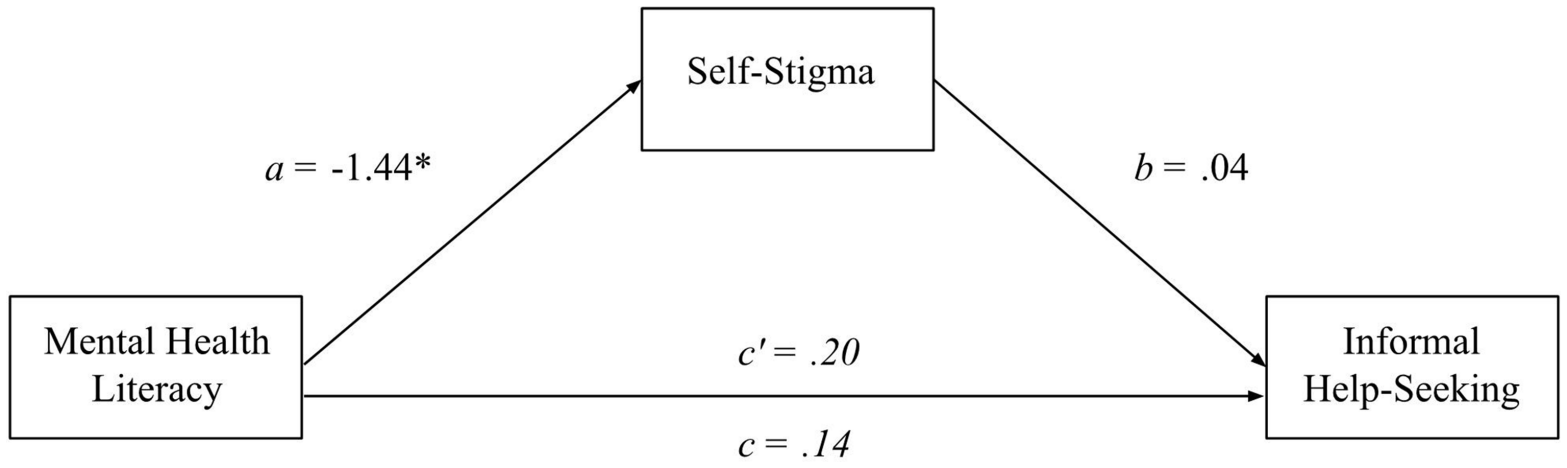
A mediation model of MHL, self-stigma, and informal help-seeking. This mediation model depicts informal help-seeking intentions as a function of MHL and self-stigma when self-stigma is controlled for (*c’*) and uncontrolled for (*c*). Furthermore, it shows the direct relationships of self-stigma to MHL and informal help-seeking. Lettered are the unstandardized beta coefficients for each relationship.

Parallel analyses as above were conducted to assess the hypothesized relationship between MHL and informal help seeking, and whether self-stigma mediated this relationship. However, the results revealed that MHL was not a significant predictor of informal help-seeking (H1b not supported), *β = 0.47, B = 0.14, t(299) = 0.80, SE_B_* = 0.18, *p* = 0.419. Although MHL was inversely related to self-stigma as found in 3.2, self-stigma did not significantly predict informal help-seeking, *β = 0.04, B = 0.04, t(299) = 0.59, SE_B_ = 0.07, p = 0.555,* nor did self-stigma act as a mediator between MHL and informal help-seeking, (*β = 0.02, SE_β_ = 0.04, 95% CI_β_ = −0.09, 0.05; B = −0.06, SE_B_ = 0.11, 95% CI_B_ = −0.28, 0.15*) see Figure 3.

### 3.5 MHL, informal help-seeking and emotional intelligence (meta-mood)

**Figure 4:**
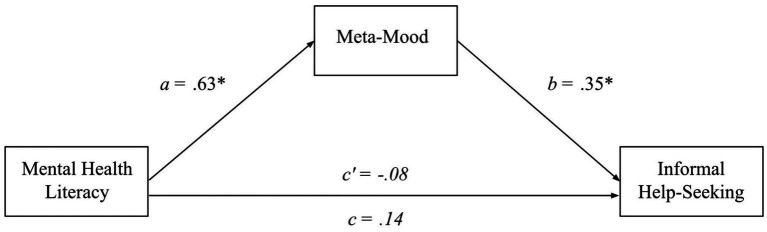
A mediation model of MHL, meta-mood, and informal help-seeking. This mediation model depicts informal help-seeking intentions as a function of mental health literacy and meta-mood when meta-mood is controlled for (*c’*) and uncontrolled for (*c*). Furthermore, it shows the direct relationship of meta-mood to MHL and informal help-seeking. Lettered are the unstandardized beta coefficients for each relationship. *p < 0.001.

Despite MHL showing no significant total effect on informal help seeking in 3.4, according to Hayes (2009), there can still be underlying mediation effects (Zhao et al., 2010). As previously identified in 3.3, MHL was a significant positive predictor of meta-mood. Additionally, meta-mood was a significant positive predictor of informal help-seeking, *β = 0.23, B = 0.35, t(299) = 3.82, SE_B_ = 0.09, p < 0.001* (see Figure 4). These relationships account for the indirect effect, bolstered by the bootstrap estimation (5,000 resamples), that meta-mood played in the relationship between MHL and informal help-seeking, (*β = 0.07, SE_β_ = 0.02, 95% CI_β_ = 0.03, 0.12; B = 0.22, SE_B_ = 0.07, 95% CI_B_ = 0.08, 0.37*). These results are consistent with an indirect-only mediation.

## 4 Discussion

The aim of the current study was to examine the relationships between MHL and formal and informal help-seeking intentions, as well as to investigate the role of self-stigma and meta-mood as possible mediators for these relationships. The results from the study are discussed below, along with implications of the findings, study limitations, and directions for future research.

### 4.1 Summary of findings

In support of H1a, it was found that MHL was a significant predictor of formal help-seeking intentions. This finding is consistent with prior research that low MHL serves as a barrier to formal help-seeking (Bonabi et al., 2016; Gulliver et al., 2010), and higher MHL is associated with higher rates of formal help-seeking (Iswanto and Ayubi, 2023; Smith and Shochet, 2011). The current study failed to establish a link between MHL and informal help-seeking intentions, contrary to H1b. This result is inconsistent with the few studies that have examined this relationship, such as Lindow et al. (2020) who found an increase in discussion about depression among peers, and in the number of students who had discussions about depression and suicidal thoughts with school staff after a high school MHL intervention. It may be that interventions targeting MHL and aimed at supporting students within a structured setting such a school environment are more likely to facilitate informal help-seeking, fostering an environment in which informal help-seeking is encouraged. In contrast, the current study examined help-seeking intentions, without the inclusion of an intervention (i.e., an actionable set of help-seeking behaviors provided to participants), which may partially explain the lack of a relationship found in the current study.

The current study’s findings, as expected, are also inconsistent with studies in which researchers used a combined measure of formal and informal help-seeking, operationalizing these as one general help-seeking construct (e.g., Smith and Shochet, 2011). Because of the differences between informal and formal help-seeking, the grouping of both types into a general help-seeking outcome may misrepresent the underlying data. D'Avanzo et al. (2012) grouped informal supports into closely related supports (friends, family, partner) and broad informal supports (clergy, teacher, someone else). These two groups of informal support displayed a disparity in terms of utilization, providing further evidence that grouping them together into a single measure in the current study may not accurately represent the underlying construct. Similarly, within the results of the current study, the results vary when separating formal and informal help-seeking, highlighting the importance of distinguishing between informal and formal supports when examining help-seeking. Especially in the context of laying the groundwork for actionable interventions, the effects of identified factors must be understood within the context of the type of help sought.

In support of H2a, it was found that self-stigma partially mediated the relationship between MHL and formal help-seeking intentions. This finding is consistent with Cawley-Fiset (2016) who found that after an MHL intervention, self-stigma towards help-seeking decreased significantly. Additionally, self-stigma has been found to be a negative predictor of formal help-seeking intentions across multiple studies (Jung et al., 2017; Fleary et al., 2022; Vogel et al., 2006; Waqas et al., 2020).

In support of H2b, meta-mood also partially mediated the relationship between MHL and formal help-seeking intentions. There is a lack of literature on meta-mood in relation to MHL; however, the current results are consistent with studies that have linked emotional intelligence to MHL. For example, emotional intelligence has been linked with higher mental illness recognition, a key facet of MHL (Furnham et al., 2011). Additionally, the link between meta-mood and formal help-seeking is consistent with related literature, which has found that higher emotional self-regulation and emotional awareness are associated with more positive attitudes towards help-seeking (Cuvalo, 2021; Komiya et al., 2000). It is important to investigate other factors involved in the relationship between MHL and formal help-seeking given that partial mediation through self-stigma and meta-mood were found in the current study.

Regarding the exploratory mediations with informal help-seeking, although the current study did not find a direct-effect relationship between MHL and informal help-seeking intentions, meta-mood did act as a mediator between the two variables. This difference in findings between formal and informal help-seeking may be due to them sharing some common mechanisms, but being conceptually distinct constructs (Saltzman et al., 2020).

Finally, it was found that meta-mood was a significant predictor of informal help-seeking intentions. While meta-mood has not been tested before in this context (to our knowledge), this finding is consistent with past literature on similar constructs. For example, emotional competency has been linked with reaching out to friends and family for support (Ciarrochi et al., 2003). Given that meta-mood was also related to aspects of MHL, a potential mechanism behind this relationship could be that while MHL concerns knowledge of mental health in general, meta-mood is the capacity of the individual to relate some of those aspects to oneself. Due to the limited literature on meta-mood, further research is necessary to establish the specific mechanisms at play.

## 5 Implications

Theoretical implications of the findings include replicating and extending the previous literature with regards to providing further support for the link between MHL and formal help-seeking and providing evidence that informal help-seeking is a distinct variable driven by different factors. These findings highlight the importance of distinguishing between the type of support rather than representing it as a general construct. From a practical perspective, not only will this allow for a better understanding of relevant factors for each type of support, but it may inform the development of more specific, and potentially effective, interventions and education. When creating interventions based on factors related to help-seeking, the knowledge that some factors such as self-stigma may act as a larger barrier to formal help-seeking than informal help-seeking can inform program development and promotion efforts.

Understanding what factors may be involved in both types of support, such as meta-mood, supports a stronger understanding of the help-seeking process and how it takes place. It may be that individuals who pay attention to their emotions recognize the importance of help-seeking in formal and informal settings more readily. This knowledge can also serve to inform evaluation of current programs involving MHL with formal help-seeking as an outcome (Fairchild et al., 2009).

The findings on informal help-seeking justify the value of assessing informal help-seeking as a distinct construct from formal help-seeking and underscores the importance of further research into predictors and mediators of informal help-seeking. By further understanding different types of help-seeking, researchers can better promote informal help-seeking within communities to support positive mental health outcomes (Levula et al., 2016), and increase formal support utilization (Fonseca and Canavarro, 2017). This is especially important in populations who may be less likely to engage in formal help-seeking behaviours (Doll et al., 2021; Zachrisson et al., 2006), as well as communities who may face more barriers to formal help-seeking (Carbonell et al., 2020; Guo et al., 2015; Vickery, 2021).

While there is some aspect of emotional intelligence involved in formal help-seeking, namely awareness and the ability to externalize and communicate the experiential feeling of mental health concerns, a parallel importance exists in informal supports (Lang, 2024). The primary component in terms of interventions seeking to promote help-seeking may benefit from focusing on awareness, and consideration of emotional states – by helping individuals better understand what they are experiencing, as well as to encouraging participants to give weight to that experience, and coupling this with an emphasis on MHL, from a help-seeking perspective, gives an individual awareness of their concern, validation that this concern is worth seeking-help for, and the knowledge necessary to engage in help-seeking behaviors (Janz and Becker, 1984; Langley et al., 2021; Kotsou et al., 2018; Wang et al., 2024).

### 5.1 Limitations and future directions

Although this study did contain some notable strengths (e.g., separating out formal and informal help-seeking into separate constructs), as with all research, there were limitations. First, the sample consisted wholly of university undergraduates taking psychology courses, most of whom were female (95%), white (40%) and young (*M* = 19.88, *95% CI = 19.46, 20.30*). This is a significant limitation because of the relationship that this sample likely has higher MHL, as psychology coursework frequently deals with mental health constructs and those enrolled in psychology courses have been shown to have higher mental health literacy when compared to other students (Lauber et al., 2005; Miles et al., 2020). Thus, this sample cannot be generalized to the public and serves to illustrate effects in the undergraduate student population. Additionally, due to the gender split, findings cannot be easily generalized to males and other genders.

To address this limitation, future research must explicitly seek out diverse populations to identify whether relevant gender, cultural, or age effects exist, as well as if demographic distributions play a role in the relationship between MHL and formal and informal help-seeking. For example, Guo et al. (2015) found that the disparity between Vietnamese-American adolescents and European-American adolescents in help-seeking behaviors was moderated by cultural factors, including perceptions of familial obligation and familial stress. Additionally, Vickery (2021) found multiple factors complicating the access of formal support for men, including challenges with disclosure and articulation of distress, and cultural norms in personal social circles. Because of these cultural influences on help-seeking among males, it is critical that future research in this area be conducted with male samples. Additionally, help-seeking research is severely lacking for populations who self-identify as gender diverse, non-binary, or gender-queer. Considering the higher risk of mental health concerns in this population, as well as disproportionately more barriers to appropriate care, there is an urgent need for research focusing on gender diverse, non-binary, and transgender populations (Chakawa, 2018; Cronin et al., 2025).

Second, the adaptation of the MHSIS into an informal help-seeking measure was not validated. It was adapted to serve as an informal alternative to mirror the formal help-seeking items; however, because it is evident that formal and informal help-seeking are related but distinct constructs, this may have been an inappropriate adaptation. Furthermore, the conceptualization of informal help-seeking as a unidimensional construct was not based on the literature. Given the variance found in the current study across specific informal supports, it may not be appropriate to have grouped informal supports together to create a single score for each participant for the purposes of the analyses (D'Avanzo et al., 2012; Lauzier-Jobin and Houle, 2022).

Finally, as mentioned previously, in addition to further investigations of informal help-seeking as its own construct, future research should explore predictors and mediators of informal help-seeking. This is important to develop an understanding of informal help-seeking and the ways in which it is similar and distinct from formal help-seeking, as well as to build an actionable understanding of both constructs to inform the creation of effective evidence-based interventions to promote informal and formal help-seeking.

## 6 Conclusion

Help-seeking is an important action individuals can take to address their mental health concerns (Yonemoto and Kawashima, 2023). To promote help-seeking, an understanding of support sources is necessary. While individuals can seek help from both formal and informal sources, the literature primarily focuses on formal supports. A need to understand the dynamics driving predictors of informal help-seeking is necessary given its importance in promoting formal help-seeking and bypassing structural barriers that individuals face in accessing formal supports. The findings of the current study illustrate a link between MHL and formal help-seeking and identify the roles of self-stigma and meta-mood in explaining this link. Although the study did not establish a relationship between MHL and informal help-seeking, it did find that MHL was related to meta-mood, and that meta-mood was related to informal help-seeking, providing key directions for future research. In conclusion, this study demonstrates the need to incorporate multiple factors when studying MHL and help-seeking outcomes, for both research and application.

## Data Availability

The raw data supporting the conclusions of this article will be made available by the authors, without undue reservation.
